# In Vivo Evaluation of a Gastro-Resistant HPMC-Based “Next Generation Enteric” Capsule

**DOI:** 10.3390/pharmaceutics14101999

**Published:** 2022-09-21

**Authors:** Adrian Rump, Marie-Luise Kromrey, Eberhard Scheuch, Vincent Jannin, Lara Rehenbrock, Mladen Vassilev Tzvetkov, Werner Weitschies, Michael Grimm

**Affiliations:** 1 Department of Biopharmaceutics and Pharmaceutical Technology, University of Greifswald, 17489 Greifswald, Germany; 2 Department of Diagnostic Radiology and Neuroradiology, University Hospital Greifswald, 17475 Greifswald, Germany; 3 Department of Clinical Pharmacology, University Hospital Greifswald, 17487 Greifswald, Germany; 4 Lonza Capsules & Health Ingredients, 68000 Colmar, France

**Keywords:** enteric hard capsule, hydroxypropyl methyl cellulose, hydroxypropyl methyl cellulose acetate succinate, MRI, caffeine, saliva, stable isotope

## Abstract

Many orally dosed APIs are bioavailable only when formulated as an enteric dosage form to protect them from the harsh environment of the stomach. However, an enteric formulation is often accompanied with a higher development effort in the first place and the potential degradation of fragile APIs during the coating process. Ready-to-use enteric hard capsules would be an easily available alternative to test and develop APIs in enteric formulations, while decreasing the time and cost of process development. In this regard, Lonza Capsugel^®^ Next Generation Enteric capsules offer a promising approach as functional capsules. The in vivo performance of these capsules was observed with two independent techniques (MRI and caffeine in saliva) in eight human volunteers. No disintegration or content release in the stomach was observed, even after highly variable individual gastric residence times (range 7.5 to 82.5 min), indicating the reliable enteric properties of these capsules. Seven capsules disintegrated in the distal part of the small intestine; one capsule showed an uncommonly fast intestinal transit (15 min) and disintegrated in the colon. The results for this latter capsule by MRI and caffeine appearance differed dramatically, whereas for all other capsules disintegrating in the small intestine, the results were very comparable, which highlights the necessity for reliable and complementary measurement methods. No correlation could be found between the gastric residence time and disintegration after gastric emptying, which confirms the robust enteric formulation of those capsules.

## 1. Introduction

The oral route is frequently used to dose active pharmaceutical ingredients (API). However, several APIs become inactive in the acidic conditions present in the stomach. Examples of this category of APIs range from small molecules (e.g., esomeprazole or erythromycin) to live biotherapeutic products (LBPs). Furthermore, APIs composed of amino acids (e.g., peptides and proteins) can be degraded by the proteolytic enzymes of the stomach [1]. Such APIs need a gastro-resistant dosage form from which the payload is released only in the milder environment of the intestine. These systems must stay intact during the gastric residence but must disintegrate and release the API in the small intestine. The delivery of the payload further down in the distal intestine would also enable the protection of the API from the enzymatic environment of the proximal intestine where most of the protease, lipase, amylase, and nuclease activities take place. This would allow protecting a larger range of fragile APIs, also including nucleic acid therapeutics, for example [2,3].

The enteric properties of oral dosage forms are generally obtained by depositing pH-sensitive polymers by coating on the dosage form or on the fill formulation. The enteric polymers classically used are insoluble in the acid pH of the stomach and readily dissolve in the milder or neutral pH of the small intestine. For example, many drugs available on the market use coated pellets to protect the API they contain from stomach acid. The development and scale-up of the coating process can be complex and generally use solvent and heat that can be detrimental to some fragile APIs (e.g., some LBPs are sensitive to dioxygen dissolved in water). A ready-to-use enteric hard capsule could be an elegant strategy to screen the usefulness of enteric formulations for new APIs, and also to minimize the risk and costs of coating processes for the development of enteric drug products. This could also be superior to coated pellets for the production of smaller batches of a product or patient-specific production, e.g., in a local pharmacy. The newly developed “Next Generation Enteric” (NGE) capsules composed of Hypromellose (HPMC) and HPMC Acetate Succinate (HPMC-AS) are a promising approach for this goal. DRcaps^®^ designed release capsules are regularly used for enteric purposes [4], but show more time-dependent disintegration which might not be sufficient if an enteric formulation is necessary [5]. Furthermore, the NGE capsules have an improved manufacturing process compared to their predecessors, which enables an efficient industrial production.

The objective of this study was to evaluate the in vivo performance of these HPMC-based NGE capsules in order to confirm the gastro-resistance of the dosage form and to assess the section of the intestine in which the capsules start to disintegrate. There are different ways to detect the gastrointestinal transit of a dosage form, i.e., the labeling with olive oil and application of an mDIXON sequence [6]. However, for this study, two independent and established methods were used, that have already proven their capabilities [5]. Magnetic Resonance Imaging (MRI) observation and caffeine detection in saliva were used as they offer complementary advantages. Indeed, a compact powder mixture with a certain proportion of ferrimagnetic black iron oxide generates a susceptibility artifact that is visible by MRI. This solid artifact has a very characteristic shape and is created when a certain amount of iron oxide powder is locally concentrated in a filled and closed capsule. The disintegration of the capsule and subsequent emptying of a proportion of the fill formulation induces a change of the spatial distribution of the ferrimagnetic particles that leads to a change of the artifact’s shape or size or even disappearance, which can be seen in MR images. Since a change in the spatial distribution of the iron oxide artifact suggests a pronounced disruption of capsule integrity, the start of the disintegration time is sometimes overestimated. It also needs to be mentioned that the viscosity of the surrounding media needs to be low enough for the iron oxide to distribute in order to be observed by MRI. Based on the above, a second independent technique to estimate the start of the disintegration time of the capsule was selected: the caffeine detection in saliva. Caffeine was selected for its high solubility and dissolution rate that enable fast dissolution when in contact with gastrointestinal media and rapid absorption in the small intestine. This leads to a caffeine appearance in saliva after its release in the small intestine in less than a minute [7]. The caffeine appearance in the saliva can also be caused by even small ruptures in the capsule, through which even small amounts of content can get into the surrounding medium, or liquid can penetrate, which then dissolves and flushes out the caffeine. Hence, disintegration evaluated by caffeine appearance correlates with the time point from which the capsules integrity is disrupted, in a way that its infill can get in contact with the surrounding media or vice versa. These two complementary techniques have already proven their value to evaluate immediate release capsules [8] as well as DUOCAP^®^ combinations of capsules [5]. However, this is the first time these techniques were used to assess the performance of gastro-resistant capsules in fasted conditions.

## 2. Materials and Methods

### 2.1. Study Materials

The capsules were provided by Lonza Capsules & Health Ingredients, Colmar, France. All capsules were filled with a powder mixture; the ingredients are listed in Table 1. Each capsule contained 25 mg ^13^C-caffeine, 13.3 mg black iron oxide, 35 mg croscarmellose, and 216.7 mg standard capsule filling powder consisting of 99.5% mannitol and 0.5% silicon dioxide. The powder components were mixed and homogenized. All capsules were filled by hand on a laboratory scale to the target fill weight of 290 mg. Due to the low bulk density of the powder filling, the capsules had a lower density than water, so they floated in water, which is typical for hard capsules. The capsules were tested without any post-filling treatment (no sealing, banding, nor coating).

### 2.2. Study Participants

Eight healthy volunteers (three males and five females) were recruited for the study. They were checked for in- and exclusion criteria and written informed consent was obtained from all subjects, including consent for the MRI measurements, handling of personal data, and confirmed German laws of data protection. Subjects had a mean age of 27.1 ± 3.3 years and a mean BMI of 22.5 ± 2.1 kg/m^2^.

### 2.3. Experimental Design

The study was performed as an open-label, single-center study. It was checked and approved by the ethical review board at the University of Greifswald, Germany (ethical protocol No. BB 180/20a) before the study participants were recruited. The study was registered at the German Clinical Trials Register with code DRKS00029609.

The subjects arrived at the study unit in the morning after at least 10 h fasting overnight. The time point t = 0 min is defined as the intake of the capsule. Two baseline saliva samples were taken at time points t = −5 min and t = −1 min and an MRI scan was completed to ensure an empty stomach (due to fasting period) at time point t = −2 min. The capsules were taken by the volunteers in an upright position in front of the MR scanner together with 240 mL of water at time point t = 0 min. Abdominal images were taken before intake of the capsule (t = −2 min), and at the following time points: 15 min, 30 min, 45 min, 60 min, 75 min, 90 min, 105 min, 120 min, 135 min, 150 min, 165 min, 180 min, 195 min, 210 min, 225 min, and 240 min. Image acquisition could be terminated as soon as the pictures showed an undoubtedly disintegrated capsule. If no disintegration was observed after 240 min, additional imaging could be performed with the additional consent of the subjects in intervals of 30 min until visible disintegration.

Saliva samples were collected one minute after each MRI measurement. After the completion of the MRI recordings, saliva samples were taken at 30 min intervals over a period of, in total, 480 min after ingestion of the capsule. By using a stable-isotope labeled caffeine species (^13^C_3_-labeled caffeine), the otherwise necessary caffeine abstinence could be waived in the study design. This makes the trial easier for the volunteers and minimizes the risk of exogenous contamination with caffeine that might affect the measurements, which in turn assures a properly performed study.

### 2.4. Salivary Sample Preparation and Evaluation of Caffeine Pharmacokinetics

The salivary sample preparation and subsequent analysis were validated and performed under good laboratory practice (GLP) conditions. Approximately 1 mL of saliva was obtained per time point and frozen at −80 °C as soon as possible; at the latest, by the end of the complete sampling period. In preparation for analysis, the saliva samples were thawed, centrifuged (15 min, 18,000× *g*), precipitated by adding 200 µL of 94% acetonitrile and 6% formic acid to 100 µL of saliva, and then frozen again. Samples were again thawed and centrifuged (15 min, 18,000× *g*). An LC-MS/MS system was used, consisting of Agilent 1100 series HPLC system (Agilent Technologies, Waldbronn, Germany), and a triple quadrupole mass spectrometer type API4000 QTRAP (AB Sciex, Darmstadt, Germany) using electrospray ionization source Turbo V™. The components were operated by the validated Analyst 1.6 software (AB Sciex, Darmstadt, Germany). This method met the criteria of the FDA Guidance for Industry “Bioanalytical Method Validation”. A detailed description of the analytical method is given in a previous publication [9]. The first time point with a measured concentration ≥12 ng/mL (lower limit of quantification (LLOQ)) of ^13^C_3_-labeled caffeine in undiluted saliva was considered as caffeine appearance.

### 2.5. Magnetic Resonance Imaging Sequences

**Figure 1:**
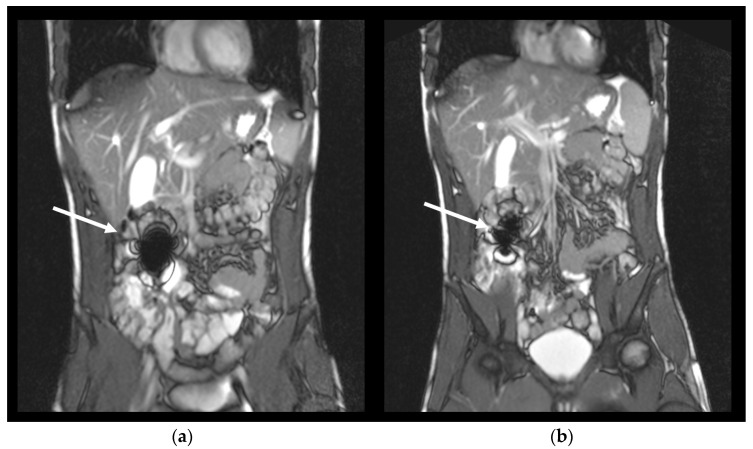
Exemplary coronal images of one volunteer with an intact capsule represented by an intact susceptibility artefact of iron oxide at 75 min after capsule ingestion in the small intestine (**a**), and the same artefact after disintegration of the capsule at 90 min (**b**). Arrows show the respective artefact.

The investigations were performed using a Siemens MAGNETOM Avanto MR-scanner (Siemens Healthcare, Erlangen, Germany) with a field strength of 1.5 Tesla in the Institute of Diagnostic Radiology and Neuroradiology. All measurements were performed in the supine position (subject lying on the back, head forward). A T2-weighted TRUFI sequence was used (Table 2). The susceptibility artifact of iron oxide can be detected clearly with this TRUFI sequence as shown in Figure 1. Transversal and coronal image slices were obtained. Subjects were asked to hold their breath for up to 23 s for each image set to reduce motion artifacts.

### 2.6. Image Analysis

An image analysis was performed using Horos Viewer Version 3.3.6 (The Horos Project). Tracking, assignment to the gastrointestinal compartments, and evaluation of disintegration time points were performed manually. All recordings were independently evaluated by three independent observers, and unclear findings were discussed.

In the MRI, capsule disintegration was observed as spreading of the characteristically shaped susceptibility artifact in the GI tract. The reduction of the artifact´s size or the appearance of several artifacts were also rated as a disintegration of the capsule, since for this to happen a capsule needed to be disintegrated to such an extent that iron oxide can spread in the surrounding media.

### 2.7. Capsule Evaluation Criteria

The gastric residence time (GRT) was defined as the mean of the last time point the capsule was located in the stomach and the first time point the capsule was located in the small intestine. The section of the gastrointestinal tract in which the capsule was located at the time of the detected disintegration was assessed as the site of disintegration. The gastric residence time and the site of disintegration were determined by MRI only, and the disintegration time was additionally determined by the first appearance of caffeine in saliva.

The disintegration time of the capsules (DT) was determined as the mean between the first measurement time point the disintegration was observed, and the last time point before this observation. Disintegration time was determined from the MRI measurements (DT_MRI_) and from ^13^C-caffeine appearance in saliva (DT_CAF_). The intestinal transit time of the capsules until disintegration (ITT_D) was determined as the difference between the gastric residence time (GRT) observed by the MRI, and both the disintegration time observed by the MRI (ITT_D_MRI_) and the disintegration time observed by the appearance of caffeine in saliva (ITT_D_CAF_).

### 2.8. Statistical Analysis

Disintegration times were given as individual data and mean ± standard deviation. The statistical evaluations were performed using Excel 2019 and OriginPro 8.5.1.

## 3. Results and Discussion

All subjects were able to swallow all capsules, and no subject experienced any adverse effects related to the study procedure.

Individual transit data and disintegration times as determined by the MRI and caffeine appearance are shown in Table 3. Average data are stated, too. The mean GRT was 43 ± 30 min (range 7.5 to 82.5 min). None of the capsules showed any sign of disintegration in the stomach. According to the MRI, the capsules disintegrated in three subjects in the jejunum and in four subjects in the ileum. In one subject, the capsule reached the ascending colon within 45 min after ingestion and disintegrated according to the MRI there 142.5 min after ingestion (DT_MRI_ = 142.5 min, ITT_D_MRI_ = 120 min).

The capsule that reached the colon showed the shortest disintegration time for caffeine (DT_CAF_ = 37.5 min). At this time point, the capsule passed the ileocecal valve. In this case, the small intestinal transit time was exceptionally fast (15 min), and therefore obviously not long enough for the capsule to disintegrate. At the first time point that the capsule was observed in the colon (45 min after ingestion), salivary ^13^C-caffeine was already detectable, indicating that the capsule had released caffeine and was therefore no longer completely intact. After the capsule reached the colon, it most likely faced a highly viscous environment, less mechanical stress/movement, and higher pH than in the small intestine. Therefore, it is likely that the capsule disintegrated to some extent when passing into the colon (at least to such an extent that caffeine was released), but the iron oxide stayed as a bulk due to the mentioned conditions and could therefore not be distinguished from an artefact of an intact capsule.

We consider ITT_D to be very suitable for evaluating the capsule performance. As the gastro-resistant capsules are monolithic dosage forms, gastric emptying in a fasted state depends on the so-called “house-keeping waves”, i.e., the third phase of the inter-digestive migrating motor complex (IMMC). The cycle length of the IMMC is typically 90 to 120 min until it starts from the beginning [10]. If only the disintegration time after capsule intake is considered, the physiology driven gastric emptying time dominates the disintegration time, whilst the ITT_D can be seen as a parameter describing the disintegration properties of the capsules. Due to the phases of the IMMC, the variability in GRT is high, as is typical for a monolithic dosage form that does not disintegrate within the stomach [11,12]. If low variability in gastric emptying is mandatory, enteric-coated pellets might be preferable.

**Figure 2:**
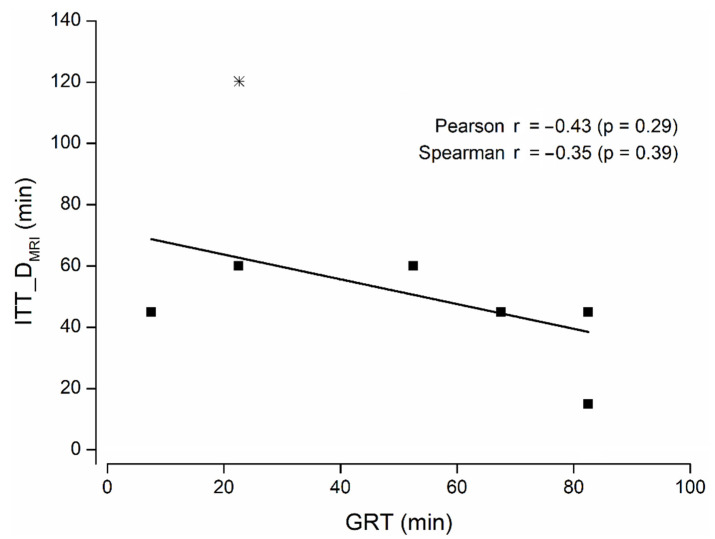
ITT_D by MRI related to GRT (n = 8). Capsule disintegration in the colon is highlighted as a star (*). Correlation is marked by a bar; correlation coefficients and corresponding *p*-values are stated.

**Figure 3:**
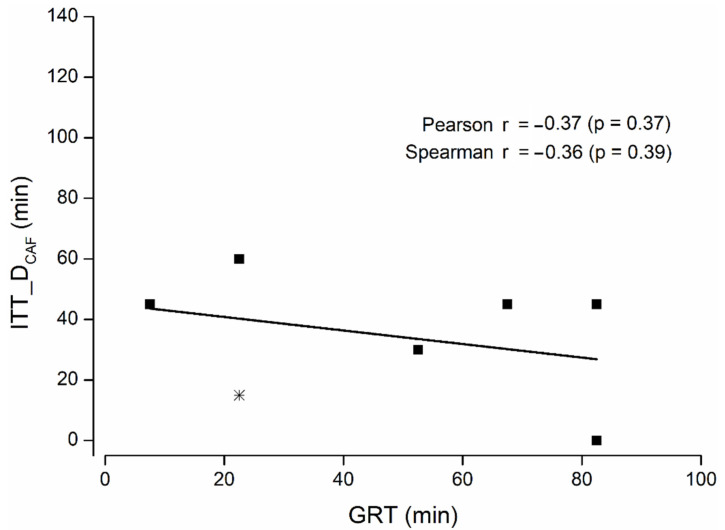
ITT_D by caffeine appearance related to GRT (n = 8). Capsule disintegration in the colon is highlighted as a star (*). Correlation is marked by a bar; correlation coefficients and corresponding values are stated.

No significant correlation between gastric residence time and intestinal transit time until disintegration was observed with either method (Figure 2 and Figure 3), indicating that the gastric residence time had no influence on the intestinal disintegration properties of the capsules. Comparable results were obtained if the colon disintegration was excluded from the evaluation.

**Figure 4:**
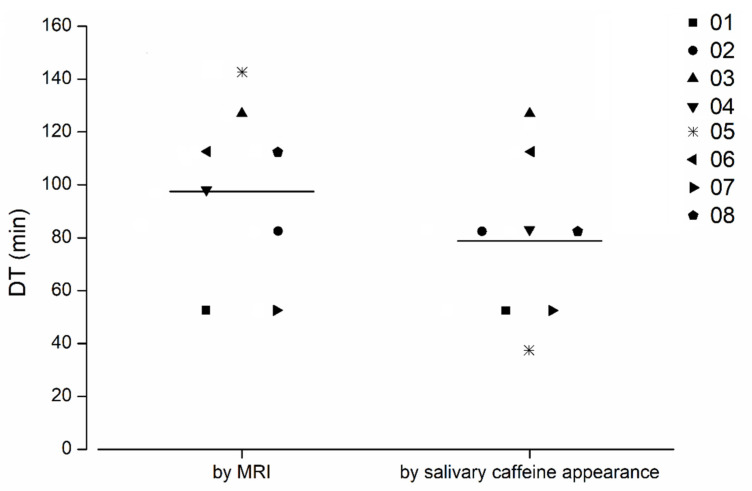
Disintegration time (DT) as determined by MRI and caffeine appearance (n = 8). Capsule disintegration in the colon is highlighted as a star (volunteer 05); the mean disintegration time is marked by a horizontal bar.

**Figure 5:**
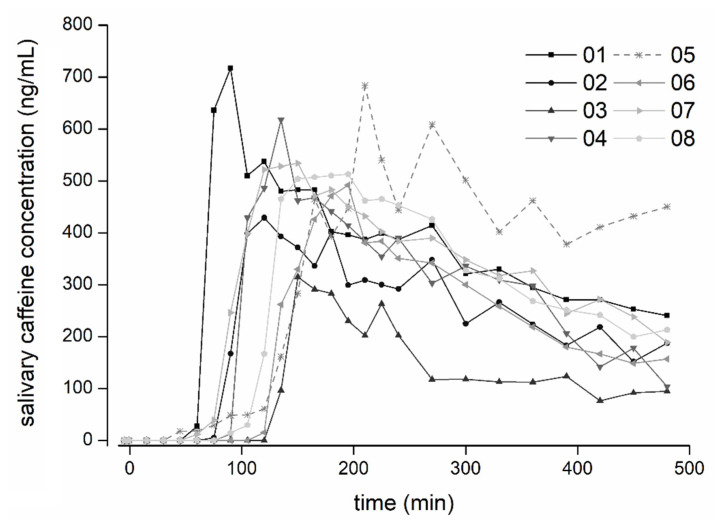
Salivary caffeine concentrations of all subjects. Profile of the capsule that disintegrated in the colon is additionally highlighted by a dashed line (volunteer 05).

For all capsules, except the one that disintegrated in the colon, the disintegration times determined by appearance of ^13^C-caffeine in saliva were identical or slightly earlier compared to the disintegration times determined by the MRI. The comparison of total disintegration times as determined by the MRI and caffeine appearance is depicted in Figure 4, and the individual caffeine profiles of all subjects are shown in Figure 5. Both detection methods provided very comparable results and confirmed a robust enteric formulation. This should be emphasized, despite the interesting finding of an extraordinarily short oro-caecal transit in one subject. This in turn highlights the importance of choosing reliable measuring methods.

## 4. Conclusions

In this study, the in vivo performance of Lonza Capsugel^®^ Next Generation Enteric (NGE) capsules was evaluated in eight healthy subjects after intake in a fasted state using MRI and ^13^C-caffeine labeling as two independent methods. Both methods showed independently robust gastro-resistant and enteric disintegration properties of the investigated hard capsules.

## Figures and Tables

**Table 1:** Ingredients of the investigated capsules.

Ingredient	Producer/Distributor
NGE size 0	Lonza, France
Black iron oxide E172	Caesar & Loretz GmbH, Hilden, Germany
^13^C_3_-labeled caffeine	SIGMA-ALDRICH CHEMIE GmbH, Schnelldorf, Germany
Croscarmellose, Ph.Eur.	JRS Pharma GmbH & Co. KG, Rosenberg, Germany
Mannitol, Ph.Eur.	Fagron GmbH & Co. KG, Barsbüttel, Germany
Silicon dioxide, Ph.Eur.	Fagron GmbH & Co. KG, Barsbüttel, Germany

**Table 2:** T2-weighted TRUFI sequence.

Parameter	Coronary Sequence Settings	Transversal Sequence Settings
Repetition time	3.4 ms	3.24 ms
Echo time	1.43 ms	1.37 ms
Slice thickness	5.0 mm	5.0 mm
Interslice gap	0.0 mm	0.5 mm
Voxel size	3.11 mm^3^	3.28 mm^3^
Flip angle	63°	63°

**Table 3:** Individual disintegration site, GRT, DT, and ITT_D for MRI and caffeine appearance. Mean values (±SD) are stated below.

	Disintegration Site	GRT (min)	DT_MRI_ (min)	DT_CAF_ (min)	ITT_D_MRI_ (min)	ITT_D_CAF_ (min)
Subject 1	Ileum	7.5	52.5	52.5	45	45
Subject 2	Ileum	22.5	82.5	82.5	60	60
Subject 3	Ileum	82.5	127.5	127.5	45	45
Subject 4	Jejunum	82.5	97.5	82.5	15	0
Subject 5	Cecum	22.5	142.5	37.5	120	15
Subject 6	Jejunum	67.5	112.5	112.5	45	45
Subject 7	Jejunum	7.5	52.5	52.5	45	45
Subject 8	Ileum	52.5	112.5	82.5	60	30
**Mean**	**-//-**	**43**	**98**	**79**	**54**	**36**
**SD**	**-//-**	**30**	**31**	**29**	**28**	**18**

## Data Availability

Data are contained within the article.
